# Deep Kernel for Genomic and Near Infrared Predictions in Multi-environment Breeding Trials

**DOI:** 10.1534/g3.119.400493

**Published:** 2019-07-09

**Authors:** Jaime Cuevas, Osval Montesinos-López, Philomin Juliana, Carlos Guzmán, Paulino Pérez-Rodríguez, José González-Bucio, Juan Burgueño, Abelardo Montesinos-López, José Crossa

**Affiliations:** * Universidad de Quintana Roo, Chetumal, Quintana Roo, 77019 México; † Facultad de Telemática, Universidad de Colima, C. P. 28040, Edo. de Colima, México; ‡ International Maize and Wheat Improvement Center (CIMMYT), Carretera Mexico- Veracruz Km. 45, El Batán, 56237, Texcoco, Edo. de Mexico, Mexico; § Colegio de Postgraduados, Montecillos 56230, Edo. de Mexico, Mexico, and; ** Departamento de Matemáticas, Centro Universitario de Ciencias Exactas e Ingenierías, (CUCEI), Universidad de Guadalajara, Guadalajara, Jalisco, 44430

**Keywords:** Genomic based prediction, Genomic Best Unbiased Predictor (GBLUP, GB linear and non-linear kernel methods), near infrared (NIR) high-throughput phenotype, single-environment model, deep learning, genomic × environment interaction model, Genomic Prediction, GenPred, Shared Data Resources

## Abstract

Kernel methods are flexible and easy to interpret and have been successfully used in genomic-enabled prediction of various plant species. Kernel methods used in genomic prediction comprise the linear genomic best linear unbiased predictor (GBLUP or GB) kernel, and the Gaussian kernel (GK). In general, these kernels have been used with two statistical models: single-environment and genomic × environment (GE) models. Recently near infrared spectroscopy (NIR) has been used as an inexpensive and non-destructive high-throughput phenotyping method for predicting unobserved line performance in plant breeding trials. In this study, we used a non-linear arc-cosine kernel (AK) that emulates deep learning artificial neural networks. We compared AK prediction accuracy with the prediction accuracy of GB and GK kernel methods in four genomic data sets, one of which also includes pedigree and NIR information. Results show that for all four data sets, AK and GK kernels achieved higher prediction accuracy than the linear GB kernel for the single-environment and GE multi-environment models. In addition, AK achieved similar or slightly higher prediction accuracy than the GK kernel. For all data sets, the GE model achieved higher prediction accuracy than the single-environment model. For the data set that includes pedigree, markers and NIR, results show that the NIR wavelength alone achieved lower prediction accuracy than the genomic information alone; however, the pedigree plus NIR information achieved only slightly lower prediction accuracy than the marker plus the NIR high-throughput data.

In genomic selection (GS) (Meuwissen *et al.* 2001), Bayesian models were introduced in the context of whole-genome regression and since then have become common in genomic prediction (Gianola 2013). Genomic-assisted selection has the advantage over phenotypic selection that it saves time and resources when making selection by adopting predictive methods and models for complex traits, along with information on pedigree, dense molecular markers and, when available, climatic covariates (de los Campos *et al.* 2009; Crossa *et al.* 2017).

Using dense molecular markers leads to the problem of n (observations)≪p (markers), which can be avoided by assuming that the contribution of molecular markers is a random variable with some distributions and with a variance-covariance matrix that consists of a scalar variance component and a known covariance kernel obtained from the markers (Gianola and Van Kaam 2008; de los Campos *et al.* 2010). This covariance kernel can model genetic effects as additive, dominant, and epistatic, as a mixture of these effects or even as genetic and non-genetic remaining effects (Crossa *et al.* 2010; Technow *et al.* 2012). During the last years, genomic-enabled predictions have usually been made with models and methods that take into account genomic × environment interaction (GE) (Burgueño *et al.* 2012; Jarquín *et al.* 2014; López-Cruz *et al.* 2015).

A variety of multi-environment models and methods can be described based on kernel matrices, and using information on genotypes, molecular markers and environmental covariables (Burgueño *et al.* 2012; Jarquín *et al.* 2014; Souza *et al.* 2017; Cuevas *et al.* 2016, 2017, 2018; Granato *et al.*, 2018). Multi-environment kernels can be constructed depending on the choice of covariance function and the multi-environment model. Usually two kernel functions have been used for genomic-enabled prediction of multi-environment breeding trials: the Genomic Best Linear Unbiased Predictor (GBLUP or GB) and the Gaussian Kernel (GK) (Cuevas *et al.* 2016 and 2017). The GB is the standard linear kernel derived from the properties of a multivariate normal distribution in linear mixed models and is usually called the genomic relationship matrix obtained from the marker matrix. The GK appeared as a reproducing kernel in the semi-parametric model Reproducing Kernel Hilbert Spaces (RKHS) (Gianola and Van Kaam 2008; de los Campos *et al.* 2010; González-Camacho *et al.* 2012) with a bandwidth (*h*) parameter that controls the rate of decay of the covariance between genotypes. In this context, Pérez-Elizalde *et al.* (2015) proposed an empirical Bayes method for estimating the bandwidth parameter *h*. Results have consistently shown for single-environment models as well as for GE models, that GK performs better than GB in terms of genomic-enabled prediction accuracy (Cuevas *et al.* 2016, 2017, 2018; Souza *et al.* 2017).

The GK kernel is one of the covariance matrices that attempts to capture complex marker effects and their possible interactions. The GK kernel is constructed based on a squared distance between a pair of genotypes multiplied by an estimated bandwidth parameter, *h*. Wilson (2014) pointed out that the potential of the non-parametric or semi-parametric Bayesian models is better expressed on big data sets. These authors mentioned that the best proposition for the Bayesian non-parametric and/or semi-parametric models is the GK kernel, which in reality does not capture all the available information existing in the data because it uses only one value for the bandwidth parameter, which is globally calculated with the available training data (marker and phenotypic data) using cross-validation methods or the marginal maximum likelihood. The consequence of having models with a tendency toward fitting global effects is that they lose the ability to account for local effects, thus limiting their predictive ability. One way to minimize this problem is to use a matrix of bandwidth values of order equal to the marker matrix; however, its estimation by cross-validation (or by any other method) is not computational feasible due to its potentially extremely large dimensions.

There have been alternatives for increasing the prediction ability of genetic values of these kernel methods; for example, Jiang *et al.* (2018) model the local epistatic effects with different kernels constructed by means of haplotypes. A kernel of different characteristics was proposed by Cho and Saul (2009) that emulates the artificial neural networks. To achieve this, the structure of the covariance matrix is constructed with a similarity matrix between genotypes that takes the Arc-cosine angle between the vector of genotypes. This makes it possible to generate a non-linear Arc-cosine kernel (named AK) that emulates the artificial neural network with one layer (Neal 1996). Cho and Saul (2009) used the theoretical framework of the kernels as the interior product of characteristic functions to emulate the hidden layers of an artificial neural network such that the kernel at one level is a function of the kernel at a previous level. Thus, the result mimics a multiple hidden layer structure (or levels) that can be used with the Bayesian paradigm from a Gaussian process with the machine learning infrastructure.

In general, kernel methods have been perceived as being much less flexible than neural network methods (Cho and Saul 2009). That is why Cho and Saul (2009) investigated the use of kernel methods in deep learning by proposing a family of kernel functions that mimic the computation of large neural networks and used those kernel functions that exploit the advantages of deep learning. The authors proposed a kernel function based on the magnitude of two observations, $x_{i}$, $x_{i^{’}}$, and the angle between them, with the main purpose of emulating the performance of neural networks with one layer. Among the basic functions, Cho and Saul (2009) proposed one function that is not linear with similar properties and functions of other kernels. Cho and Saul (2009) also proposed a recursive formula to emulate the performance of various layers (*l*), that is, the covariance matrix (kernel) in one layer (or level) is computed by taking a function of the previous kernel, and applying it to classification problems with a multilayer kernel machine algorithm. The new kernel methods developed by Cho and Saul (2009) had distinct properties, and the authors evaluated them on a wide range of real data sets and observed important increases in accuracy, computer speed and memory consumption.

Machine learning is a field of computer science that has the ability to improve performance on a specific task from data. In general, machine learning explores algorithms that can learn from current data and make predictions on new data, by building a model from sample inputs (Samuel 1959). Several machine learning algorithms uses an artificial neural network with multiple layers linked nonlinearly. The “deep” in deep learning refers to the number of layers through which the data are transformed. The layers in these methods consist of multiple stages of nonlinear data transformations, and the features of the data are represented by successively higher layers in the neural network system. In plant breeding, initial applications of artificial neural networks were used for genomic-enabled prediction (González-Camacho *et al.* 2012, 2016; Ornella *et al.* 2012, 2014). The application of new deep learning methods for genomic-enabled prediction was recently studied for extensive maize and wheat data sets including or not including GE, and for continuous as well as mixed binary, ordinal and continuous multi-traits and multi-environments (Montesinos-López *et al.* 2018a,b and 2019a,b). Results show that artificial neural networks in the form of one or more hidden layers (deep machine learning) could be an efficient option for handling big and complex data sets that are common in plant breeding.

It has been shown that other sources of molecular variation can be used for the prediction of complex traits; however, these tools have limited applications in large plant breeding trials. Recently, researchers have used low cost non-destructive tools such as near-infrared spectroscopy (NIR) and nuclear magnetic resonance as a high-throughput phenotype for predicting grain yield and other end-quality traits in various crop species (Hayes *et al.* 2017; Rincent *et al.* 2018). Interestingly, Rincent *et al.* (2018) used high-throughput phenotyping for wheat lines and a tree species (poplar) irradiated with infrared measured absorbance from 400 to 2500 nm. Rincent *et al.* (2018) used NIR wavelength data as regressors to calculate the NIR similarity matrix between genotypes (NIR BLUP) in grains and leaves, and found better prediction accuracy than when using molecular markers, GBLUP (genomic similarity matrix). Hayes *et al.* (2017) used a multi-trait approach that incorporates NIR and nuclear magnetic resonance information in the genomic models for end-use quality traits in wheat. The authors found that genomic predictions ranged from 0-0.47, whereas after adding information using NIR and nuclear magnetic resonance, prediction accuracy increased to 0-0.69.

Based on the above statistical and biological considerations and in an attempt to examine the performance of new kernel methods in genomic-enabled prediction, we used the recursive formula of Cho and Saul (2009) for constructing the arc-cosine kernel (AK), where the kernel in one layer (or level) is a function of the kernel in the previous layer. The genomic-enabled prediction accuracy of AK is compared with the accuracy of GB and GK kernels in a single-environment as well as GE models. The AK is incorporated as a kinship or covariance matrix of the random effects of a genomic mixed model (de los Campos *et al.* 2010). The number of levels (layers, *l*) to be used is defined by optimizing the marginal maximum likelihood on the training set, which is similar to what Pérez-Elizalde *et al.* (2015) proposed for computing the bandwidth parameter *h* of the Gaussian Kernel. We compared the prediction accuracy of the Arc-cosine Kernel (AK) *vs.* GB and GK using genomic data and for one data set, using genomic, pedigree and NIRs data. The comparisons between genomic-enabled prediction accuracy between the different methods (GB, GK and AK) were performed using random cross-validations that form different random groups of training (TRN) and testing (TST) sets, and computing the correlations between the observed and the predictive values for each random partition. Thus, the average correlations from all partitions and their standard deviations are calculated and reported. To form the TRN and TST sets in models including GE, the random cross-validation CV2 is used where a certain percentage of the lines are unobserved and thus predictive in some environments but observed in other environments (Burgueño, *et al.* 2012).

For this study, we used 4 data sets. Three data sets with only genomic data (one wheat, WHEAT1, and two maize, MAIZE2 and MAIZE3 data sets) were already published; we fitted these three data sets with models including single-environment and GE multi-environment models with the three kernels methods: GB, AK, and GK. We also used an unpublished wheat data set from the CIMMYT Global Wheat Program (WHEAT4) comprising elite wheat lines evaluated in one environment and genotyped with dense molecular markers. For data set WHEAT4, the wheat seed of the lines incorporated information on NIR. For these data sets, prediction accuracy combining pedigree, markers, and NIR was studied using only the single-environment model with the GB, GK and AK kernels.

## Material and Methods

In this study, we used the same models (single-environment and GE models) and methods (GB and GK) on all four data sets (WHEAT1, MAIZE2, MAIZE3, and WHEAT4) including non-linear kernel AK in all data sets plus pedigree and NIR information in the WHEAT4 data set. We briefly define methods for GB and GK kernels and describe the new AK non-linear kernel in detail.

### Kernel methods

The GBLUP (GB) is the standard linear kernel derived from the properties of a multivariate normal distribution in linear mixed models and is usually called the genomic relationship matrix obtained as $\text{GB}=\frac{XX^{\prime }}{p},\;$where ***X*** could be the scaled marker matrix (with *p* molecular markers) (López-Cruz *et al.* 2015) or the kernel matrix formed based on NIRs (NIR BLUP). The GK appeared as a reproducing kernel in the semi-parametric model Reproducing Kernel Hilbert Spaces (RKHS) (González-Camacho *et al.* 2012). It is defined as $\text{GK}=\exp\left(-hd_{ii^{’}}^{2}/q\right),$ where *h* is the bandwidth parameter that controls the rate of decay of the covariance between genotypes, and q is the median of the square of the Euclidean distance, $d_{ii^{’}}=\underset{k}{\sum }{\left(x_{ik}-x_{i^{’}k}\right)}^{2}$, which is a measure of the genetic distance between individuals based on molecular markers. Pérez-Elizalde *et al.* (2015) proposed an empirical Bayes method for estimating the bandwidth parameter *h*.

Below we describe the Arc-cosine kernel (AK) method proposed by Cho and Saul (2009) that includes one basic function or level (layer one) and a recursive function for different levels of recursion.

### Arc-cosine Kernel (AK)

This method considers that the main similarity measure between two individuals is the angle between two vectors that is computed from marker genotypes (or NIRS) $x_{i}$, $x_{i^{’}}$, that is:

$$\;\;\theta_{i,i^{\prime }}=\cos^{-1}\left(\frac{x_{i}\cdot x_{i^{\prime }}}{\left\|x_{i}\right\|\left\|x_{i^{\prime }}\right\|}\right)$$

where ⋅ denotes the inner product and $\left\|x_{i}\right\|$ is the norm of line *i*. Neal (1996) showed that the following kernel is semidefinite positive and related to the neural network with a single layer.

$${\text{AK}}^{1}\left(x_{i},x_{i^{\prime }}\right)=\frac{1}{\pi }\left\|x_{i}\right\|\left\|x_{i^{\prime }}\right\|J\left(\theta_{i,i^{\prime }}\right)$$ (1)

where π is the pi constant (3.1415...) and J(θ)=[sin(θ)+(π−θ)cos(θ)]. Equation (1) gives a symmetric positive semidefinite matrix ($AK^{1}$ with entries given in Equation 1) preserving the norm of the entries such that $\text{AK}\left(x_{i},x_{i}\right)={\left\|x_{i}\right\|}^{2}$, and $\text{AK}\left(x_{i},-x_{i}\right)=0$ and models non-linear relationships.

Because the kernel is the inner product in an induced characteristic space $\text{Φ}(x_{i})\cdot \text{Φ}(x_{i^{’}})$, the reasoning of Cho and Saul (2009) is that repeating this process l times $\text{Φ}(\text{Φ}\left(...\text{Φ}\left(x_{i}\right)\right))\cdot \text{Φ}(\text{Φ}\left(...\text{Φ}\left(x_{i^{’}}\right)\right))$ will emulate the performance of the neural network with *l* hidden layers by using the following recursive formula.

$$\begin{array}{l} AK^{\left(l+1\right)}(x_{i},x_{i^{’}})={\frac{1}{\pi }[AK^{\left(l\right)}\left(x_{i},x_{i}\right)AK^{\left(l\right)}\left(x_{i^{’}},x_{i^{’}}\right)]}^{\frac{1}{2}}J\left(\theta_{i,i’}^{\left(l\right)}\right)\\ \theta_{i,i’}^{\left(l\right)}=cos^{-1}\{AK^{\left(l\right)}(x_{i},x_{i^{’}}){\left[AK^{\left(l\right)}\left(x_{i},x_{i}\right)AK^{\left(l\right)}\left(x_{i^{’}},x_{i^{’}}\right)\right]}^{-\frac{1}{2}}\} \end{array}$$ (2)

Equation (1) considers the case with l=1 (AK^1^), from where (2) is derived for l>1 (AK^2^, AK^3^ ...). Note that (1) does not require continuous bandwidth as in the case of GK (Gaussian Kernel). Also from (2), the *l* level with the best predictive ability can be selected using the training set and the recursive equation. We selected the *l* level that has the maximum marginal likelihood in a way that is similar to what Pérez-Elizalde *et al.* (2015) did to select the bandwidth parameter (*h*) of the GK kernel method.

### Statistical models

The statistical models employed in this study were defined and described in Cuevas *et al.* (2016, 2017, 2018) and used by Souza *et al.* (2017) for the single-environment model and the GE model using the GB, and the GK kernel methods. A brief discussion is given below.

### General linear model

The following general mixed linear model can cover a great diversity of cases and models such as those described in Crossa *et al.* (2010), Jarquín *et al.* (2014), López-Cruz *et al.* (2015), and Crossa *et al.* (2017) for relating the phenotypic observations in one environment or in multi-environments, with covariables like dense molecular markers, pedigree, environmental covariables, NIR, and others.

$$y=\mu 1+X_{f}\beta +\overset{q}{\underset{r=1}{\sum }}u_{r}+\varepsilon$$ (3)

where ***y*** is the vector of observations of size *n*, the scalar *μ* is a general mean, matrix $X_{f}$ is an incidence matrix for fixed effects ***β***. It is assumed that the random vectors $u_{r}\;(r=1,...q)$, each of size *n*, are independent from other random effects and follow a normal distribution $u_{r}∼N(0,\sigma_{u_{r}}^{2}K_{r})$, with $\sigma_{u_{r}}^{2}$ being a variance component parameter estimated by the model, and $K_{r}$ a known semidefinite positive symmetric matrix of order n×n. The random errors are assumed independent with normal distributions $\varepsilon ∼N\left(0,\sigma_{\varepsilon }^{2}I\right),$ where $\sigma_{\varepsilon }^{2}$ is the error variance.

### Single-environment model

For a single environment and only one kernel, equation 3 can be expressed as:

$$y=\mu 1+X_{f}\beta +u+\varepsilon$$

where vector ***y*** represents the phenotypic observations in that environment, ***u*** is the genomic random effects assumed normally distributed as $u∼N(0,\sigma_{u}^{2}K)$, where $\sigma_{u}^{2}$ is the genomic variance estimated from the data, matrix ***k*** is constructed as $K=Z_{g}GZ_{g}^{'}$ where matrix $Z_{g}\text{ relates }\;$the genotypes with the observations, ***G*** models the relationship of the genotypes and could be a pedigree matrix or a genomic matrix or a matrix based on NIR (Rincent *et al.* 2018) throughout GB, GK and AK, as previously described. The random errors are assumed independent with normal distributions $\varepsilon ∼N(0,\sigma_{\varepsilon }^{2}I)$, where $\sigma_{\varepsilon }^{2}$ is the error variance.

### Multi-environment models

In the GE multi-environment reaction norm model (Jarquín *et al.* 2014; López-Cruz *et al.* 2015; Cuevas *et al.* 2016), equation (3) takes the form

$$y=\mu 1+X_{f}\beta +u_{1}+u_{2}+\varepsilon ,$$

where $y={\left[y_{1},...,y_{m}\right]}^{'}$ are the observations in each of the *m* sites (or environments). If environments are considered fixed effects, they are represented in the ***β*** effects and included in matrix $X_{f}$ , which relates the sites (or environments) to the phenotypic observations. For convenience, in the notations we call this matrix $Z_{e}$ (Souza *et al.* 2017), that is, $X_{f}=Z_{e}$. In this model, $u_{1}∼N(0,\sigma_{u_{1}}^{2}K_{1})$ represents the genomic main effects, $\sigma_{u_{1}}^{2}$is the genomic variance component estimated from the data, and $K_{1}=Z_{g}GZ_{g}^{'}$, where $Z_{g}$ relates the genotypes to the phenotypic observations. The random effects representing the interaction between the genomic effects and their interaction with sites or environments are modeled as $u_{2}∼N(0,\sigma_{u_{2}}^{2}K_{2})$, where $K_{2}=(Z_{g}GZ_{g}^{'}){}^{\circ}Z_{e}Z_{e}{}^{’}$, where ° is the Hadamard (or Schur) product. The Hadamard product takes two matrices of the same order and generates a matrix as a result of multiplying each element (i,j) from one matrix with the corresponding element (i,j) of the other matrix (of the same order). As previously mentioned, matrix $Z_{e}$ relates the sites (or environments) to the phenotypic observations.

### Method for selecting the number of layers (l) for the AK kernel

The following sequence is used to select the appropriate *l* level (number of layers) when fitting the AK kernel with the single-environment and the GE multi-environment models.

- The first step is computing the ***G*** base matrix for l=1 using equation (1) from the markers or the NIR, such that $AK^{1}=G$ (single-environment and GE multi-environment) (see the auxiliary function in link hdl.handle.net/11529/10548180 where the four data sets and other codes are given).
- Compute the maximum marginal likelihood for this first level using the available training data.
  $$m\left(y\right)=\int_{0}^{\infty }\overset{\infty }{\underset{0}{\int }}\overset{\infty }{\underset{-\infty }{\int }}p(u,\sigma_{u}^{2},\sigma_{\varepsilon }^{2}|y)dud\sigma_{\varepsilon }^{2}\;d\sigma_{u}^{2}$$
  This is similar to what Pérez-Elizalde *et al.* (2015) did when estimating the bandwidth parameter *h* for the GK kernel (see the APPENDIX).
- Construct $AK^{l+1}$ using equation (2).
- Compute the maximum (*M*) marginal (*M*) likelihood of $AK^{l+1}$, $MML_{\left(l+1\right)}$.
- Compute the maximum marginal likelihood of $AK^{l},$ $AK^{l+1}$; if maximum marginal (MM) $MML_{\left(l+1\right)}$ > $MML_{\left(l\right)}$, we increase one level l=l+1 and repeat from c); if $MML_{\left(l+1\right)}$ <= $MML_{\left(l\right)}$, we select l.

With the selected level of *l* we compute the kernel matrices required for the model and thus fit the model. To select the level of *l* for the GE multi-environment model, we only consider the main effects, which is where there is exchange (borrowing) of information between environments. That is, when modeling GE, we use phenotypic observations from all environments; thus, when deriving the prediction for a given line not observed in one environment, the GE model benefits from records of that line that were collected in other correlated environments. This is the random cross-validation CV2 (López-Cruz *et al.* 2015) that considers some lines observed in some environments and predicted in others.

### Phenotypic and genotypic data sets

#### WHEAT1:

This data set was first used by Crossa *et al.* (2010) and also by Cuevas *et al.* (2016, 2017) and comprises 599 wheat lines from the CIMMYT Global Wheat Program evaluated in four international environments representing four basic agroclimatic regions (mega-environments). The phenotypic trait considered here was grain yield (GY) of the 599 wheat lines evaluated in each of the four mega-environments. The 599 wheat lines were genotyped using 1447 Diversity Array Technology (DArT) markers generated by Triticarte Pty. Ltd. (Canberra, Australia; http://www.triticarte.com.au). The markers were filtered and those with a minor allele frequency lower than 0.05 were removed, and missing genotypes were imputed using samples from the marginal distribution of marker genotypes. The number of DArT markers after filtering was 1279.

#### MAIZE2:

This data set was included in Souza *et al.* (2017) and is from the Helix Seeds Company (HEL). It consists of 452 maize hybrids obtained by crossing 111 pure lines (inbreds); the hybrids were evaluated in 2015 at five Brazilian sites (E1-E5). The experimental design used in each site was a randomized block with two replicates per hybrid. The parent lines were genotyped with an Affymetrix Axiom Maize Genotyping Array of 616 K SNPs (Single Nucleotide Polymorphism) (Unterseer *et al.* 2014). Standard quality controls (QC) were applied to the data, removing markers with a *Call Rate* ≥ 0.95. The remaining missing data in the lines were imputed with the Synbreed package (Wimmer *et al.* 2015) using the algorithms from the Beagle 4.0 software (Browning and Browning 2009). Markers with Minor Allele Frequency (MAF) of ≤ 0.05 were removed. After applying QC, 52,811 SNPs were available to make the predictions. The phenotypic and genomic data of inbred lines are credited to Helix Seeds Ltda. Company.

#### MAIZE3:

This maize data set was included in Souza *et al.* (2017), comes from USP (Universidad Sao Paulo) and consists of 740 maize hybrids obtained by crossing 49 inbred lines. The hybrids were evaluated in four environments (E1-E4) in Piracicaba and Anhumas, São Paulo, Brazil, in 2016. The hybrids were evaluated using an augmented block design, with two commercial hybrids as checks to correct for micro-environmental variation. At each site, two levels of nitrogen (N) fertilization were used. The experiment conducted under ideal N conditions received 100 kg ha^-1^ of N (30 kg ha^-1^ at sowing and 70 kg ha^-1^ in a coverage application) at the V8 plant stage, while the experiment with low N received 30 kg/ha of N at sowing. As in the MAIZE1 data set, the parent lines were genotyped with an Affymetrix Axiom Maize Genotyping Array of 616 K SNPs. Markers with Minor Allele Frequency (MAF) of ≤ 0.05 were removed. After applying QC, 54,113 SNPs were available to make the predictions.

#### WHEAT4:

A total of 561 elite wheat lines from CIMMYT’s advanced bread wheat breeding program were evaluated in field trials for grain yield and genotyped with 5,500 molecular markers. The collected 1059 NIR wavelengths were normalized by taking the first (NIR1) and second derivative (NIR2) in an attempt to increase the possible differences in sample signals that might be overlooked without this mathematical treatment. The pedigree information was extracted and used for prediction based on pedigree by using the numerical relationship matrix ***A***. The single-environment model was also fitted using the numerical relationship matrix ***A*** in combination with the NIR BLUP and the genomic similarity between wheat lines given by the linear ***G*** kernel of the GBLUP.

### NIR (Near-infrared reflectance spectroscopy) used for the WHEAT4 data set

Spectroscopy in the near infrared has been used widely for the rapid determination of organic components. The NIR technique requires that the energy absorbed in the near-infrared region by a sample causes covalent bonds of C-H, O-H, and N-H, important components of organic substances, to vibrate in different forms. For this study we used an NIRS 6500 FOSS-NIR System that poses a scanner software ISIScan version 1.6 on the range of wavelength from 400 to 2500 nanometers (nm) of reflectance. The database was managed by the software WinISI and the reflectance data were stored as the log (1/R) (where R is the reflectance) using intervals of 20 nm. We deposited samples consisting of 20-25 grams of clean wheat seed in the cell for the NIR.

### Assessing prediction accuracy by random cross-validation

In each data set we partitioned the data 50 times, with 70% of the data for training and 30% for testing. The prediction accuracy was measured by Pearson’s correlation between the predicted and observed values (the standard deviation was also computed). For data sets WHEAT1, MAIZE2, and MAIZE3, the predictions were made for each environment for both models (single-environment model and GE multi-environment model) using kernels GB, GK and AK constructed with the molecular markers. For model GE multi-environment, the random cross-validations formed a CV2 cross-validation where some lines had been tested in some environments but not in others (Burgueño *et al.* 2012). For data set WHEAT4, we compared results only for the single-environment model for kernels GB, GK and AK from the markers and from the NIRs obtained with the first and second derivatives. We also included the pedigree and fitted different models with different combinations of markers, NIR and pedigree.

In data set WHEAT1, we made predictions using AK1-AK4 with the objective of comparing the prediction accuracy of AK with different numbers of levels (layers, *l*). In all the data sets we computed the appropriate number *l* using the marginal maximum likelihood. When fitting each model, we used 30,000 iterations, eliminated the first 5000 and thinned every three iterations.

### Software

Models were implemented using R codes (R Core Team 2018). In particular for the first and second derivatives of the matrix of NIRs of WHEAT4 data set, we used the Savitzky Golay function from the library prospector (Stevens and Ramirez-Lopez 2014). For fitting the models and making predictions, we used the BGGE function from the BGGE library (Granato *et al.* 2018). As mentioned in Granato *et al.* (2018), the BGGE is an algorithm constructed to fit models considering a Bayesian framework that uses the Gibbs sampler for the Montecarlo Markov Chain (MCMC), which allows convergence to a posterior predictive distribution that provides the predictive values. The codes can be found at link hdl.handle.net/11529/10548180.

The link has word files identified as WHEAT1, MAIZE2 MAIZE3, and WHEAT4 data sets explaining where the different data sets are located, where the R codes are located, and the name of the files where the R codes of the methods (GB, GK, AK) are and where the models (single-environment) and GE environment are placed.

### Data availability

Although WHEAT1, MAIZE2, and MAIZE3 data sets have been published and the data are already available from those articles, we have uploaded them again at the following link, together with WHEAT4 data (pedigree, markers and NIRs). At this link hdl.handle.net/11529/10548180 we have included the phenotypic and genotypic data of each data set so that the readers can download and use it immediately without having to do any further data manipulation, extraction, etc. Furthermore, the folders of each data set contain all the R software used to analyze each data set, as previously described [there are two files with the R codes for computing the various functions, Auxiliar.functions.R for AK and GE, and another one that is used for each data set with all the models named Model.(WHEAT1…)R. In addition, we added a word file explaining all the previous files].

## Results

Results are presented by data set for the single-environment and GE multi-environment models and for the three kernel methods, GB, AK and GK. The tables present the average correlation between the observed and predictive values for each model-method. The best predictive correlations are in boldface for the corresponding method (AK; GK; GB). Tables 1-5 have two types of superscripts; (+) indicates the significant *t*-test of the hypothesis that the average correlation of the AK method is higher at the 5% probability level than the average correlation of the GB method. The superscript (*) denotes the significant *t*-test of the hypothesis that the average correlation of the AK method is higher at the 5% probability level than the average correlation of the GK method.

### WHEAT1 data set

**Figure 1:**
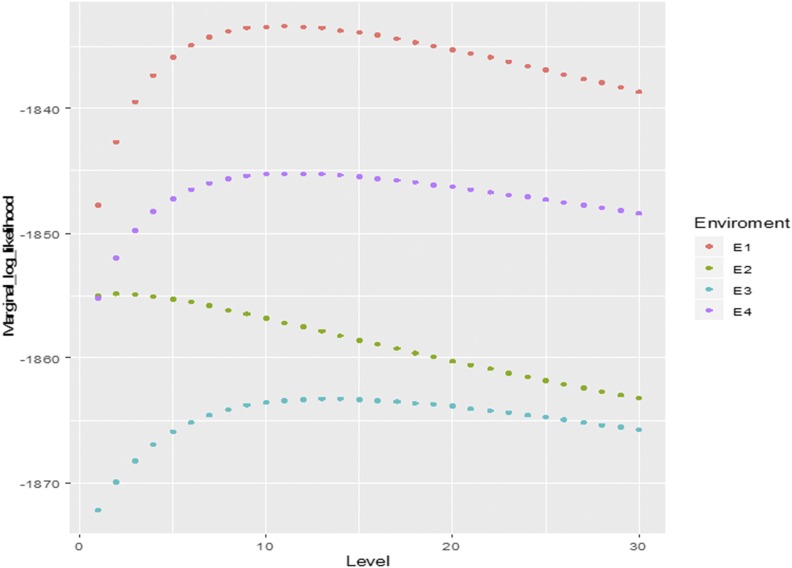
WHEAT1 data set. Marginal likelihood for several levels (layers) for environments.

The highest genomic-enabled prediction accuracies for the single-environment model were obtained with the AK method, closely followed by the GK method (Table 1). The genomic prediction accuracy of various AK methods (AK^1^ – AK^4^) increased as more levels (layers) were added, but their accuracy did not improve after four layers, whereas the AK with the recursive method for increasing the number of layers by examining the marginal likelihood achieved higher prediction accuracy than the AK^1^ – AK^4^ methods. Table 1 shows the levels (layers) of the maximum marginal likelihood for each environment; these values are also displayed in Figure 1 for each environment (E1-E4). The prediction accuracy of the AK method was significantly higher than that of the GB method but not of the GK method.

**Table 1:** WHEAT1 data set. Average Pearson’s correlations between observed and predictive values (and their standard deviation in parentheses) for seven methods for a single-environment model for 50 random partitions with 70% of the lines in the training set and 30% of the lines in the testing set. Methods GB, GK, and AK are the GBLUP, Gaussian Kernel, and Arc-Cosine Kernel, respectively. Methods ${\text{AK}}^{1}$ - ${\text{AK}}^{4}$ correspond to the Arc-Cosine kernel model with 1-4 levels (layers). The best predictive model for each environment (E1-E4) is in boldface

Environment	GB	${\text{AK}}^{1}$	${\text{AK}}^{2}$	${\text{AK}}^{3}$	${\text{AK}}^{4}$	AK	Level	GK
Single-environment model								
E1	0.490 (0.04)	0.520 (0.04)	0.536 (0.04)	0.544 (0.04)	0.551 (0.04)	**0.561****+** (0.04)	11	**0.561** (0.04)
E2	0.469 (0.05)	0.474 (0.05)	0.476 (0.05)	0.477 (0.05)	**0.478** (0.05)	**0.478** (0.05)	3	0.477 (0.05)
E3	0.378 (0.06)	0.390 (0.05)	0.400 (0.05)	0.401 (0.05)	0.409 (0.05)	**0.419****+** (0.04)	13	0.416 (0.05)
E4	0.450 (0.05)	0.470 (0.05)	0.482 (0.05)	0.491 (0.04)	0.491 (0.04)	**0.508****+** (0.05)	11	0.506 (0.05)

^+^ Significant at the 0.05 probability level of the t-test for the hypothesis that the average of the correlation of kernel AK is superior to the mean of the correlation of kernel GB.

When the GE multi-environment model was fitted, the method with the highest accuracy was AK, again closely followed by the GK method (Table 2) (no significant differences). Except for the genomic-enabled prediction of E1 (that has negative correlations with the other 3 environments, Burgueño *et al.* 2012), where the single-environment model was more accurate than the GE multi-environment, for the other environments (E2-E4), the GE multi-environment had higher prediction accuracy than the single-environment model for all three methods GB, AK and GK. The GB kernel method did not overcome any of the two nonlinear kernel AK and GK in terms of prediction accuracy. For the GE multi-environment model, six levels (layers) were selected using the previously described marginal likelihood.

**Table 2:** WHEAT1 data set. Average Pearson’s correlations between observed and predictive values (and their standard deviation in parentheses) for three methods for a GE multi-environment model for 50 random partitions with 70% of the lines in the training set and 30% of the lines in the testing set. Methods GB, GK, and AK are the GBLUP, Gaussian Kernel and Arc-Cosine Kernel, respectively, with six levels (layers). The best predictive model for each environment (E1-E4) is in boldface

Environment	GB	AK	Level	GK
GE multi-environment model				
E1	0.422 (0.05)	**0.494****+** (0.06)	6	0.482 (0.06)
E2	0.537 (0.04)	**0.582****+** (0.04)	6	0.581 (0.04)
E3	0.441 (0.05)	**0.497****+** (0.06)	6	0.494 (0.05)
E4	0.485 (0.05)	**0.558****+** (0.04)	6	0.551 (0.05)

^+^ Significant at the 0.05 probability level of the t-test for the hypothesis that the average of the correlation of kernel AK is superior to the mean of the correlation of kernel GB.

### MAIZE2 data set

In general, the prediction accuracies for the MAIZE2 data set were higher than those found in the WHEAT1 data set. The best predictive method was AK, which had significantly higher prediction accuracies than the GK and GB methods for all single-environment analyses (Table 3). The differences between the AK and GK methods for E1-E5 were substantial and significant in terms of average correlation (*e.g.*, E1:755 AK *vs.* 0.733 GK; E2: 0.533 AK *vs.* 0.488 GK; E3: 0.759 AK *vs.* 0.718 GK; E4: 0.502 AK *vs.* 0.469 GK; E5: 0.564 AK *vs.* 0.522 GK).

**Table 3:** MAIZE2 data set. Average Pearson’s correlations between observed and predictive values (and their standard deviation in parentheses) for 3 methods for single-environment and GE multi-environment models for 50 random partitions with 70% of the lines in the training set and 30% of the lines in the testing set. Methods GB, GK, and AK are the GBLUP, Gaussian Kernel and Arc-Cosine Kernel, respectively. The best predictive model for each environment (E1-E5) is in boldface

Single-environment model			
Environment	GB	AK	GK
E1	0.647 (0.07)	**0.755**+* (0.05)	0.733 (0.05)
E2	0.384 (0.07)	**0.533**+* (0.06)	0.488 (0.07)
E3	0.678 (0.03)	**0.759**+* (0.02)	0.718 (0.04)
E4	0.368 (0.03)	**0.502**+* (0.06)	0.469 (0.05
E5	0.393 (0.07)	**0.564**+* (0.06)	0.522 (0.06)

GE multi-environment model			
E1	0.679 (0.08)	**0.811****+** (0.03)	0.810 (0.05)
E2	0.476 (0.09)	**0.600****+** (0.05)	**0.600** (0.06)
E3	0.694 (0.05)	**0.784****+** (0.03)	**0.784** (0.03)
E4	0.409 (0.09)	0.547**+** (0.05)	**0.550** (0.06)
E5	0.478 (0.09)	0.643**+** (0.04)	**0.644** (0.04)

* Significant at the 0.05 probability level of the t-test for the hypothesis that the average of the correlation of kernel AK is superior to the mean of the correlation of kernel GK.

^+^Significant at the 0.05 probability level of the t-test for the hypothesis that the average of the correlation of kernel AK is superior to the mean of the correlation of kernel GB.

When the GE multi-environment model was fitted, results show that the highest prediction accuracies for grain yield were obtained with AK in environment E1 (0.811); similar predictions were achieved with AK and GK in E2 and E3, and GK had a higher average correlation to AK in environments E4 and E5 (Table 3). In all the environments, the prediction accuracies achieved by the GE multi-environment model were higher than those obtained by the single-environment model with all three methods. Also, the non-linear kernels significantly outperformed the linear kernel GB in all five environments (Table 3).

### MAIZE3 data set

This data set has four environments, and for the single-environment model, non-linear kernels AK, GK and linear kernel GB gave very similar (and non-significantly different) prediction accuracies; AK accuracies ranged from 0.271 (E1) to 0.421 (E4) (Table 4), whereas GK accuracies ranged from 0.273 (E1) to 0.423 (E4).

**Table 4:** MAIZE3 data set. Average Pearson’s correlations between observed and predictive values (and their standard deviation in parentheses) for 3 methods for single-environment and GE multi-environment models for 50 random partitions with 70% of the lines in the training set and 30% of the lines in the testing set. Methods GB, GK, and AK are the GBLUP, Gaussian Kernel and Arc-Cosine Kernel, respectively. The best predictive model for each environment is in boldface

Single-environment model			
Enviroment	GB	AK	GK
E1	0.246 (0.05)	0.271^+^ (0.05)	**0.273** (0.05)
E2	**0.321** (0.04)	0.319 (0.04)	0.320 (0.04)
E3	**0.295** (0.06)	0.294 (0.06)	0.294 (0.06)
E4	0.42 (0.05)	0.421 (0.05)	**0.422** (0.05)

GE multi-environment model			
E1	0.301 (0.05)	**0.520**^+^ (0.05)	0.517 (0.05)
E2	0.342 (0.04)	**0.505**^+^ (0.05)	0.497 (0.04)
E3	0.330 (0.05)	**0.478**^+^ (0.04)	0.470 (0.04)
E4	0.435 (0.05)	**0.555**+* (0.04)	0.546 (0.05)

* Significant at the 0.05 probability level of the t-test for the hypothesis that the average of the correlation of kernel AK is superior to the mean of the correlation of kernel GK.

^+^Significant at the 0.05 probability level of the t-test for the hypothesis that the average of the correlation of kernel AK is superior to the mean of the correlation of kernel GB.

Regarding the results from the GE multi-environment model, the AK kernel showed a slight increase in prediction accuracy over the GK and much greater and significant prediction accuracy (average correlations) than the linear kernel GB. Also, for all combinations of environments and kernels, the prediction accuracies of the GE multi-environment were higher than those of the single-environment model, thereby reaffirming the advantages of using GE models to improve the prediction accuracy (Table 4). For the GE multi-environment model, the AK method had significantly higher average correlations between the observed and predictive values than the GB linear kernel but no significant differences with the GK non-linear method.

### WHEAT4 data set

**Figure 2:**
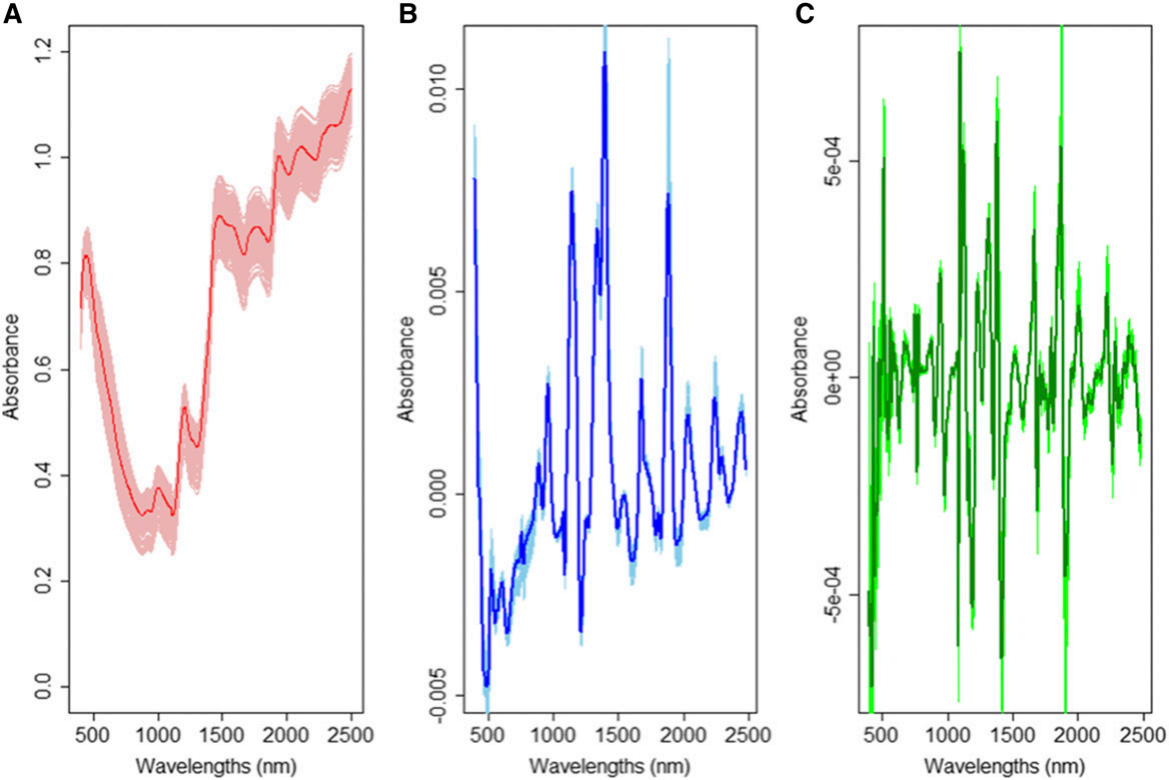
WHEAT4 data set. All spectra for wheat lines are depicted in the three figures; light colors are for each wheat line, while the strong color line in each graph is the average of all spectra. (a) absorbance raw data (red); (b) normalized absorbance (first derivative) (blue); (c) normalized second derivative (green).

This data set has only one environment, so only the single-environment model can be studied. However, information on several kernel covariance matrices is available (pedigree, marker, and NIR). In general, and across all cases, the average of the predictions of the non-linear kernel GK is similar to that of the AK kernel (Table 5); although their average correlations were higher than those of the GB methods, the statistical test does not reject the hypothesis of being similar. Figures 2a-c depict the patterns of variation of the absorbance for raw NIR, first derivative and second derivative, respectively. The first and second derivatives (NIR1 and NIR2, respectively) computed under the Savitzky and Golay (1964) method, filtered the noise of the original NIRs and generated the NIR1 and NIR2 with a more symmetric distribution around zero, thus eliminating some abnormal shapes of the original spectra (see Figure 2) that should help to improve the prediction accuracy (Rincent *et al.* 2018).

**Table 5:** WHEAT4 data set. Average Pearson’s correlations between observed and predictive values (and their standard deviation in parentheses) for 3 methods for a single-environment model for 50 random partitions with 80% of the lines in the training set and 20% of the lines in the testing set. Methods GB, GK, and AK are the GBLUP, Gaussian Kernel and Arc-Cosine Kernel, respectively. The best predictive model for each environment is in boldface. The three GB, AK, and GK were applied to data from NIR1 (first derivative), NIR2 (second derivative), markers, pedigree and some combinations. The best predictive model for each type of data used is in boldface

Data used	GB	AK	GK
NIR1	0.349 (0.07)	0.347 (0.07)	**0.354** (0.07)
NIR2	0.346 (0.07)	**0.367** (0.07)	0.354 (0.07)
GENOMIC	0.424 (0.07)	**0.456** (0.07)	0.454 (0.07)
GENOMIC+NIR1	0.436 (0.07)	**0.462****+** (0.07)	0.456 (0.07)
GENOMIC+NIR2	0.435 (0.07)	**0.466****+** (0.07)	0.455 (0.07)
Pedigree	0.396 (0.07)	—	—
GENOMIC+Pedigree	0.437 (0.07)	0.450 (0.07)	**0.454** (0.07)
Pedigree +NIR1	0.420 (0.07)	0.413 (0.07)	**0.425** (0.07)
Pedigree +NIR2	**0.421** (0.07)	0.418 (0.07)	**0.421** (0.07)
GENOMIC+Pedigree+NIR1	0.448 (0.07)	0.455 (0.07)	**0.462** (0.07)
GENEMIC+Pedigree+NIR2	0.448 (0.07)	**0.460** (0.07)	0.459 (0.07)

^+^ Significant at the 0.05 probability level of the t-test for the hypothesis that the average of the correlation of kernel AK is superior to the mean of the correlation of kernel GB.

When using only NIR information (NIR1, NIR2), the prediction accuracy is much lower than the accuracy found when using genomic-enabled for the three kernels: GB = 0.424 (GENOMIC) *vs.* 0.349 (NIR1), AK = 0.456 (GENOMIC) *vs.* 0.347 (NIR1), and GK = 0.454 (GENOMIC) *vs.* 0.354 (NIR1) (Table 5). For pedigree, the BLUP used the numerical relations matrix (***A***), which gave prediction accuracies higher than NIR1 and NIR2 but lower than genomic prediction. It is interesting to observe that NIR2 (with a second derivative) improved the prediction accuracy with respect to NIR1 with AK. Results show that combining markers with NIR (either NIR1 or NIR2) increased the prediction accuracy over the accuracy of using markers alone for all the three kernels (GB, GK, and AK). Interestingly, combining pedigree with NIR (either NIR1 or NIR2) gave slightly lower prediction accuracy compared with the prediction using genomic for the three kernels (GB pedigree+NIR1 = 0.420 *vs.* GENOMIC = 0.424; GK pedigree+NIR1 = 0.425 *vs.* GENOMIC = 0.454; AK pedigree+NIR1 = 0.413 *vs.* GENOMIC = 0.456) (Table 5). These results (NIR +pedigree) may be useful as a feasible alternative when markers are not available.

The two combinations with the highest prediction accuracy were kernel AK with GENOMIC + NIR2 (0.466) and kernel AK with GENOMIC+pedigree+NIR2 (0.460). Although the prediction accuracy of NIR2 *vs.* NIR1 did not change for kernel GK, for kernel AK, NIR2 was better than NIR1. In combination with markers, NIR2 was slightly superior to NIR1 only when used with kernel AK. In combination with pedigree, NIR2 and NIR1 did not change much when used with the different kernels.

## Discussion

The main objective of this research was to show a kernel method, the Arc-cosine, AK, as an alternative to the Gaussian kernel but avoiding the need to estimate the bandwidth, which requires intense computing resources. The AK kernel also offers an alternative to the use of deep learning that requires the definition of a large number of hyper parameters. However, in this study, we did not compare the genomic-enabled prediction accuracy between AK and deep learning.

Tables 1-4 show that the results for WHEAT1, MAIZE2, and MAIZE3 follow the same pattern, that is, non-linear methods (AK, GK) gave significantly higher prediction accuracy than the linear GB kernel, with the exception of the single environment of the MAIZE3 data set. Methods AK are GK gave no significantly different prediction accuracy except for the single-environment model, where AK was significantly better than GK and GB. Nevertheless, in the WHEAT4 data set, the hypothesis that AK is not different from GB and GK is not rejected.

In general, the accuracy of genomic prediction is influenced by: (1) linkage of extensive blocks of chromosomes from parents in the training sets that are passed on to the progeny (for example in F2 bi-parental populations where maximum linkage disequilibrium, LD, is achieved for extensive chromosome blocks), (2) linkage disequilibrium between dense markers and certain QTL that affect the trait in the training and testing sets, and (3) both linkage of extensive blocks and linkage disequilibrium. Usually in self-pollinated crops like wheat, the extent of linkage disequilibrium is important (Crossa *et al.* 2007); thus closely linked markers and QTL will remain together through a certain number of generations, and nonlinear models such as GK and AK could be suitable for capturing cryptic locally epistatic effects for prediction accuracy (Akdemir and Jannink 2015). In maize, linkage blocks are shorter than in wheat, so capturing epistatic effects should be more problematic than in wheat. Models that account for these epistatic effects are complex and must have a non-linear component to capture these small hidden relationships between markers. The GK and AK non-linear kernels were much more capable of capturing and exploiting these cryptic and small relationships than the linear kernel GB. For the WHEAT1 and MAIZE2 data sets, kernel AK was slightly but consistently superior in terms of prediction accuracy to GK for both the single-environment and the GE multi-environment models. For the MAIZE3 data set, kernel AK was consistently superior in terms of prediction accuracy to GK for the GE multi-environment models. For data set WHEAT4, there are more cases where GK was superior to the AK kernel.

### Kernels with genomic information – WHEAT1, MAIZE2, and MAIZE3 data sets

Parametric genomic models (such as the linear GB) that use kernels constructed with markers allow clear biological and genetic interpretation of the genomic estimates from marker effects and their interactions with the environment accounted for by the reaction norm models (López-Cruz *et al.* 2015). These models capture only additive genetic effects and account for additive genetic variance of the measured traits. However, their genomic-enabled prediction is usually lower than that of other non-linear kernels like GK, as demonstrated by Cuevas *et al.* (2016, 2017, 2018) and Souza *et al.* (2017). Nevertheless, models based on GB can also represent the transformation of one kernel into another kernel. For example, a polynomial kernel of order two of GB represents a non-linear relationship. Methods for transforming one valid kernel into another valid kernel can be found in Wilson (2014, p. 40).

The Gaussian kernel (GK) has been used in genomic prediction by Crossa *et al.* (2010), de los Campos *et al.* (2010), and Cuevas *et al.* (2016, 2017, 2018) and can model cryptic relationships between markers that usually improve prediction accuracy. This is partly due to the fact that matrix GK can model non-linear relationships between lines because the bandwidth parameter models these relationships as estimated in the training set through cross-validation or by maximizing the marginal likelihood (Pérez-Elizalde *et al.* 2015). However, selecting the bandwidth has two inconveniences: it requires intensive computing time and assumes that the training set can determine the true optimum value of the bandwidth. These inconveniences can be solved in part by scaling the Euclidean distances by their median (Crossa *et al.* 2010); this gives good results particularly when the markers are standardized. The similarity measurement used in GK, the squared Euclidean distance, can be interpreted as the frequency of the differences in the presence of markers. Morota and Gianola (2014) mentioned that no other kernel has higher prediction.

### The AK kernel

Results of this study show that for some data sets, the AK kernel may perform the same as or slightly better than the GK kernel. These results are of interest for exploring hidden and small marker relationships using a similarity measurement different to that employed by the GK. The AK uses the magnitude of the vectors between two lines and the angle induced by the interior product in its characteristic space; this repetition generates a recursive formula for exploring hidden relationships and simulating layers. In this study, we used equations (1) and (2) to construct the AK that maximizes the marginal distribution. Equation (1) generates the basic matrix and in practice, it consumes less computing time than the required for GK when computing the distances between individuals based on marker. The optimal level (number of layers) depends on the training data sets and varies for each data set. For example, Table 1 for the WHEAT1 data set shows that the prediction accuracy increases as more hidden layers are included, until reaching a maximum that could correspond to the level that maximizes the marginal likelihood. Figure 1 shows that for WHEAT1, the increase in prediction accuracy is related to the increase in the maximum of the marginal likelihood. Every environment can have a different pattern of marginal likelihood; for example, in E2, the maximum values of the marginal likelihood are around 3 layers, whereas in the other environments, the maximum values of the marginal likelihood are around 10 levels (layers). However, Table 2 shows that for WHEAT1 and the GE multi-environment model, the level of layers used was 6; this is the level that maximizes the marginal likelihood when considering the main effects (the optimum number of layers can change depending on whether the marker matrix is or not scaled). It should be noted that for random cross-validation CV2, the exchange (borrowing) of information between environments occurs throughout the genomic main effects. Cho and Saul (2009) proposed an algorithm where the output from one level is the input to another level, mimicking the deep learning neural network algorithm that is explained in Montesinos-López *et al.* (2018a,b; 2019a,b).

As previously mentioned, for selecting level *l*, we initially computed the marginal likelihood, moved one more level and recalculated the marginal likelihood, and continued the process until there was no further improvement in the likelihood. Thus the data are fitted with this last *l* level. There are other alternatives that could be studied, for example, considering other basic functions as $AK_{i,i^{’}}^{1}=\frac{1}{\pi }\;\left(\pi -\theta_{i,i^{’}}\right)$, as proposed by Cho and Saul (2009). Another alternative would be to fit the data with *l* = 1 and prepare the output to be used as input for the next level. We have tried this approach with varying results; nevertheless, further research is required. In the future, research is needed to compare the prediction accuracy of AK including different layers with the deep learning artificial neural network method studied by Montesinos-López *et al.* (2018 a,b); this type of study would require comparing AK (having different numbers of layers) with single-environment and GE multi-environment models with deep learning neural networks (having different numbers of layers) under both models.

### Kernels with genomic and near infrared plus pedigree information WHEAT4 data set

As proposed by Rincent *et al.* (2018), in this study we used the near infrared (NIR) method as a high-throughput method for constructing relationship matrices based on the reflectance of various wavelengths applied to sample seed of different bread wheat lines. Reflectance should be related to some chemical properties of seed samples for numerous wavelengths. Rincent *et al.* (2018) proposed evaluating the efficiency of the NIR for making predictions utilizing the NIRs as markers (NIR BLUP), similar to what the GBLUP does in conventional genomic prediction. Here the similarity between lines is given by the NIR wavelengths. Rincent *et al.* (2018) proposed using NIRs for phenomic selection defined as high-throughput phenotyping, thus obtaining a large number of variables (wavelength) as regressors in statistical models similar to those used in GS. The authors noted that as opposed to classical methodology, where NIRS collected on a sample serve to make predictions about that particular sample, their proposition is to make predictions for other samples (*e.g.*, environments). Rincent *et al.* (2018) found for several traits in wheat and a tree species (poplar) that the NIR BLUP prediction accuracies from grains, leaves, and wood are better than genomic prediction accuracy.

Our results using NIR, pedigree, and markers show that using only NIR does give similar prediction accuracy as when using pedigree, but lower prediction accuracy than when using markers. In general, the accuracies of genomic-enabled prediction observed in this study are similar to those previously reported for grain yield of CIMMYT elite wheat lines. Markers gave higher correlations between observed and predicted values than pedigree and NIR. Interestingly, pedigree + NIR gave only slightly lower prediction accuracy (0.413-0.425) than marker + NIR (0.435-0.466) and similar prediction accuracy to prediction accuracies using markers, pedigree and NIR together (0.448-0.462) (Table 5). It is likely that the main reason for this is that the wheat lines used in this study are elite lines with not much grain yield differences between them.

In other work Hayes *et al.* (2017) used genomic, NIR and nuclear magnetic resonance (NMR) for the prediction of grain and end-use traits that usually comprise low samples with small amounts of flour. Under these circumstances, it is important to use additional information to complement genomic-enabled predictions. The authors found that incorporating NIR and NMR into the genomic multi-trait model increased the accuracies from 0 to 0.47 to 0-0.69 for 19 end-use quality traits. In our study, the inclusion of NIR increased the prediction accuracy of the GBLUP genomic model, especially for the AK kernel (0.456 Pearson’s correlation for GBLUP *vs.* 0.462 and 0.66 for GBLUP+NIR1 and GBLUP+NIR2, respectively; Table 5). Interestingly, for the GK model there was almost no increase in accuracy of GBLUP *vs.* GBLUP+NIR1 and GBLUP+NIR2.

The results from the WHEAT4 data set are preliminary and used on highly elite wheat lines as compared to those of Hayes *et al.* (2017), which included highly variable wheat accessions, and the study of Rincent *et al.* (2018), which comprised a highly diverse historical panel of wheat lines from many years. However, our results agree with previous studies where the inclusion of NIR did increase the prediction accuracies of the lines in the testing set when using markers, when using pedigree and when using both. An important result is that the use of pedigree and NIR offers an opportunity to increase prediction accuracy similar to that obtained when using markers plus NIR (at a higher cost).

### Conclusions

In this study we used three kernel methods for genomic prediction: the linear genomic best linear unbiased predictor (GBLUP) kernel (GB), the non-linear Gaussian kernel (GK) and the non-linear Arc-cosine kernel (AK). The AK emulates deep learning artificial neural networks and is required to define the *l* levels that define the number of layers. The three data sets included only genomic information, whereas one data set included near infrared spectroscopy (NIR), genomic information, and pedigree information. Our study included two statistical models: the single-environment and the genomic × environment (GE) models. For the four data sets, methods AK and GK achieved higher prediction accuracy than the linear GB kernel for the single-environment model, as well as the GE multi-environment model. Interestingly, AK achieved similar or slightly higher prediction accuracy than the GK kernel. For data set WHEAT4 (with pedigree, genomic information and NIR), NIR wavelength alone achieved less prediction accuracy than the genomic information alone. Interestingly, pedigree + NIR information achieved slightly lower prediction accuracy than genomic information + NIR. We conclude that the AK kernel has the advantage over the GK, which does need to estimate the bandwidth parameter while producing similar prediction accuracies.

## Appendix

For simplicity, assume model y=u+ε, where ***y*** is centered at zero, $u∼N(0,\sigma_{u}^{2}K$) and $\varepsilon ∼N(0,\sigma_{\varepsilon }^{2}I$**)**. The marginal distribution is the integral over all the parameters (noise) of the posterior distribution:

$$m\left(y\right)=\overset{\infty }{\underset{-\infty }{\int }}\overset{}{\underset{R^{n}}{\int }}\overset{\infty }{\underset{0}{\int }}p(u,\sigma_{u}^{2},\sigma_{\varepsilon }^{2}|y)dud\sigma_{\varepsilon }^{2}d\sigma_{u}^{2}$$

$$m\left(y\right)=\int_{-\infty }^{\infty }\overset{}{\underset{R^{n}}{\int }}\overset{\infty }{\underset{0}{\int }}p(y|u,\sigma_{u}^{2},\sigma_{\varepsilon }^{2})p\left(u\right)p\left(\sigma_{\varepsilon }^{2}\right)p(\sigma_{u}^{2})dud\sigma_{\varepsilon }^{2}\;d\sigma_{u}^{2}\;$$

The first step is to integrate over vector ***u***, after eigen values decomposition of matrix ***K***, such that K=USU', where ***U*** is an orthogonal matrix of the eigen vectors and ***S*** is a diagonal matrix with the eigen values on its diagonal order from the highest to the lowest. Vector ***b*** is transformed as $b=U^{'}u$ and assuming $\sigma_{u}^{2}=\phi \sigma_{\varepsilon }^{2},$ then the distribution of ***b*** is $b∼N\left(0,\phi \sigma_{\varepsilon }^{2}S\right).$

To facilitate the computations, the integration is along ***b*** instead of ***u*****:**

$$\overset{\infty }{\underset{-\infty }{\int }}p(y|u,\sigma_{\varepsilon }^{2},\sigma_{u}^{2})p\left(u\text{|}\sigma_{\varepsilon }^{2},\sigma_{u}^{2}\right)du=\overset{\infty }{\underset{-\infty }{\int }}{\left(2\pi \sigma_{\varepsilon }^{2}\right)}^{-n/2}{\left(2\pi \sigma_{\varepsilon }^{2}\right)}^{-n/2}|\phi S{|}^{-0.5}\exp\left(-\frac{{\left(y-\mathrm{Ub}\right)}^{'}(y-\mathrm{Ub})}{2\sigma_{\varepsilon }^{2}}\right)\exp\left(-\frac{{\left(b\right)}^{'}S^{-1}(b)}{2\phi \sigma_{\varepsilon }^{2}}\right)db$$

Completing the rest of the terms are

$$=\;\frac{A}{{\left(\sigma_{\varepsilon }^{2}\right)}^{\left(n-1\right)/2}}\exp\left(-\frac{B}{2\sigma_{\varepsilon }^{2}}\right)$$

where$\;\;A=\frac{n^{0.5}\prod_{i=1}^{n}{\left(s_{i}^{2}\phi +1\right)}^{-0.5}}{{\left(2\pi \right)}^{(n-1)/2}}$ and $B=y^{'}U{\left(1+S^{-2}\frac{1}{\phi }\right)}^{-1}U^{'}y+y^{'}y$

Now if we consider that the prior distribution of $\sigma_{\varepsilon }^{2}$ is $\left(\sigma_{\varepsilon }^{2}\right)=\frac{1}{\sigma_{\varepsilon }^{2}}$, then

$$\overset{\infty }{\underset{0}{\int }}\frac{A}{{\left(\sigma_{\varepsilon }^{2}\right)}^{\frac{n-3}{2}}}\exp\left(-\frac{B}{2\sigma^{2}}\right)d\sigma^{2}$$

$m\left(y\text{|}\phi \right)=\frac{A\text{Γ}(\frac{n-1}{2})B^{-(n-1)/2}}{2^{-(n-1)/2}}\;\propto \overset{n}{\underset{i=1}{\prod }}{\left(1+\phi s_{i}\right)}^{-\frac{1}{2}}{\left[\overset{n}{\underset{i=1}{\sum }}\frac{{\tilde{d}}_{i}^{2}}{\left(1+\phi s_{i}\right)}\right]}^{-\frac{n-1}{2}}$, where ${\tilde{d}}_{i}^{2}$ is the *i*^th^ value of $\tilde{d}=U^{\prime }y$.
